# Using a MEMS All-Quartz Fiber-Optic Fabry–Pérot High-Temperature Pressure Sensor Based on a Nanoforest Array to Enhance Interference Signals

**DOI:** 10.3390/mi17101191

**Published:** 2026-10-15

**Authors:** Jun Wang, Hanzhi Lin, Jianjun Yang, Qianyu Ren, Pinggang Jia

**Affiliations:** State Key Laboratory of Widegap Semiconductor Optoelectronic Materials and Technologies, North University of China, Taiyuan 030051, China; b20210625@st.nuc.edu.cn (J.W.); s202519032@st.nuc.edu.cn (H.L.); yangjj@nuc.edu.cn (J.Y.); renqianyu@nuc.edu.cn (Q.R.)

**Keywords:** fiber-optic sensor, fiber-optic Fabry–Pérot interferometer, all-quartz structure, MEMS, nanoforest array, fiber microsphere lens

## Abstract

This paper presents and experimentally demonstrates an all-quartz fiber-optic Fabry–Pérot (FP) high-temperature pressure sensor with interference signal enhancement based on micro-electro-mechanical system (MEMS) technology. By incorporating a nanoforest array and an optical microsphere lens, the quality of the optical interference signal generated by the multilayer fiber-optic FP sensing structure is effectively enhanced. MEMS fabrication and high-temperature bonding techniques are employed to enable batch fabrication of the sensing units, while laser fusion splicing is utilized to achieve adhesive-free packaging and integration of the sensor with high-temperature-resistant optical fibers. Experimental results demonstrate that the fabricated all-quartz fiber-optic FP high-temperature pressure sensor enables stable pressure measurements over a range of 0–200 kPa within a temperature range of 20–700 °C. At room temperature, the sensor exhibits a pressure sensitivity of 56.6 nm/kPa and a maximum nonlinearity error of 0.49% F.S. In addition, the sensor maintains good operational stability under high-temperature conditions. Owing to its all-quartz structure, the proposed sensor offers excellent high-temperature resistance, good temperature stability, and strong potential for batch fabrication, providing a viable approach for the development and application of fiber-optic pressure sensors in high-temperature environments.

## 1. Introduction

Pressure measurement in high-temperature environments is of significant engineering importance in a wide range of applications, including aerospace, oil and gas exploration, chemical processing, and metallurgy [1]. Currently, sensors capable of high-temperature pressure measurement can be broadly classified into two categories: electrical and fiber-optic. Typical electrical pressure sensors include piezoelectric, piezoresistive, and capacitive devices. However, in the aforementioned application scenarios, sensors are often required to operate continuously under extreme conditions, such as under high temperatures and in highly corrosive environments. The operation of these electrical devices relies heavily on semiconductor components and nearby electronic conditioning circuits, which are highly susceptible to environmental temperature variations, thereby significantly limiting their practical applicability under extreme high-temperature conditions [2]. In contrast, fiber-optic pressure sensors employ optical signals for sensing and information transmission, while their sensing probes can be fabricated from high-temperature-resistant materials. These sensors offer several distinctive advantages, including excellent high-temperature resistance, immunity to electromagnetic interference, intrinsic safety, and resistance to chemical corrosion, and have therefore emerged as a promising research direction for high-temperature pressure measurement [3]. Extrinsic Fabry–Pérot interferometer (EFPI) pressure sensors have attracted considerable attention for pressure measurement under extreme operating conditions owing to their intrinsically decoupled and independently tunable measurement range and sensitivity, as well as their excellent structural robustness [4,5].

EFPI pressure sensors typically consist of a high-temperature-resistant sensing structure and a high-temperature-resistant optical fiber, which are responsible for pressure sensing and optical signal transmission, respectively. Their high-temperature operating capability is primarily determined by the temperature resistance of the sensing materials, the fabrication processes of the sensing structures, and the quality of the optical interference signals. To date, a variety of materials have been employed to fabricate high-temperature pressure-sensitive structures, including silicon, quartz, magnesium oxide, sapphire, and polymers [6]. Conventional EFPI pressure sensors typically form a Fabry–Pérot (FP) cavity between the inner surface of a pressure-sensitive diaphragm and the end face of an optical fiber, with the two components being interconnected through adhesive bonding, adsorption, or mechanical fixation [7,8]. However, such structures are susceptible to the thermal stability of the joining materials, interfacial reliability, and thermal expansion mismatch, which can compromise their durability at elevated temperatures. In addition, the sensing performance is highly sensitive to the cavity length and the parallelism of the interfering interfaces, making it difficult for conventional assembly processes to ensure device-to-device consistency. Furthermore, the reliable fabrication and batch production of hermetically sealed cavities capable of withstanding high temperatures remain challenging [9]. To improve structural stability, multilayer EFPI pressure sensors have attracted considerable attention. These sensors precisely define the FP cavity length through a multilayer material configuration, thereby reducing the influence of conventional assembly processes on cavity length uniformity. However, existing multilayer sensors predominantly employ multi-mode fibers (MMFs) for optical signal transmission [10], and the associated modal noise and relatively large beam size can limit the signal demodulation speed. In contrast, single-mode fibers (SMFs) offer greater transmission stability and the potential for faster response; however, their interference signals are more sensitive to the reflectivity of the interfaces [11]. When the reflectivity of the interfering surface is low, both the interference visibility and signal-to-noise ratio (SNR) are significantly reduced, thereby increasing the difficulty of signal demodulation and degrading pressure measurement accuracy. Increasing the reflectivity of the interfering interface is therefore an effective approach to improving the optical signal quality of SMF-based EFPI sensors [12]. The deposition of a high-reflectivity coating or an antireflection coating on the surface of the sensing structure is a commonly adopted strategy [13]. However, conventional thin-film materials are susceptible to insufficient thermal stability, degraded interfacial reliability, and thermal expansion mismatch at elevated temperatures, which can lead to film cracking, delamination, or drift in optical properties, thereby limiting the maximum operating temperature of some sensors to below 400 °C [14]. In contrast, quartz exhibits excellent thermal resistance, a low coefficient of thermal expansion, and superior thermal stability, making it an ideal material for constructing high-temperature EFPI pressure sensors [15,16]. Therefore, achieving reliable fabrication of multilayer EFPI structures while fully exploiting the high-temperature properties of quartz and simultaneously obtaining high-quality and stable optical interference signals remains a key challenge in developing high-performance, highly consistent, and batch-manufacturable quartz-based high-temperature pressure sensors.

In this paper, we propose and experimentally demonstrate an all-quartz fiber-optic FP high-temperature pressure sensor with interference signal enhancement based on micro-electro-mechanical system (MEMS) technology. A nanoforest structure is introduced to enhance the optical transmittance of the wafer, and computer numerical control (CNC) machining, high-temperature bonding, and laser fusion splicing are integrated to enable the batch fabrication of highly consistent and high-temperature-resistant pressure-sensitive components. Experimental results demonstrate that the fabricated sensor enables pressure measurements from 0 to 200 kPa over a temperature range of 20–700 °C. At room temperature, the sensor exhibits a pressure sensitivity of 56.6 nm/kPa, a maximum nonlinearity error of 0.49% F.S., and an FP interference peak-to-valley difference of approximately 29.22 dB. The proposed fabrication approach addresses the requirements for integrating low-reflectivity, high-temperature-resistant materials such as quartz into fiber-optic FP high-temperature pressure sensors while maintaining good high-temperature pressure measurement performance, device-to-device consistency, and optical interference signal quality. This work provides a feasible approach for achieving high-SNR signal acquisition and batch fabrication of multilayer fiber-optic FP high-temperature pressure sensors based on low-reflectivity materials.

## 2. Sensor Principle and Design

**Figure 1:**
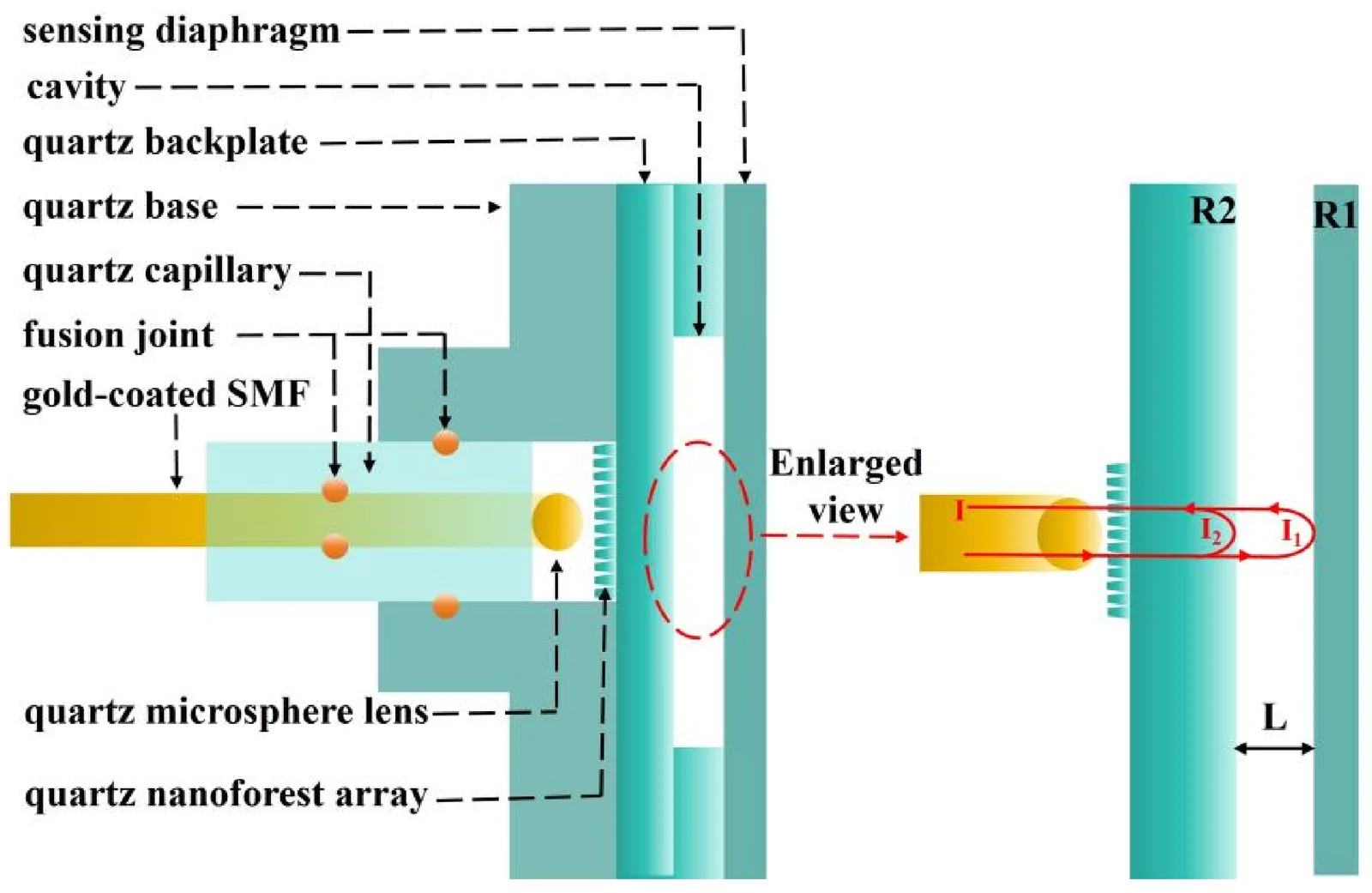
Schematic diagram of the all-quartz fiber-optic FP pressure sensor.

The all-quartz fiber-optic FP pressure sensor developed in this study primarily consists of a quartz sensitive diaphragm, a quartz cavity layer, a quartz backplate, a quartz substrate, a quartz capillary, and a quartz single-mode gold-coated optical fiber integrated with a microsphere lens, as shown in Figure 1. The quartz sensitive diaphragm is thinned using MEMS technology, while a through-hole with a uniform thickness is fabricated in the quartz cavity layer by CNC machining. A nanoforest array structure is fabricated on the outer surface of the quartz backplate, and the quartz substrate is used to connect the quartz capillary and the quartz single-mode gold-coated optical fiber. The nanoforest array can enhance the optical transmittance of the quartz wafer [17], and the single-mode gold-coated optical fiber integrated with a microsphere lens provides effective beam collimation, as demonstrated in our previous studies [18]. The inner surface of the quartz sensitive diaphragm and the inner surface of the quartz backplate form the two interference surfaces of the FP cavity, thereby forming a low-finesse FP cavity. The light emitted from the optical fiber sequentially passes through the outer surface of the backplate and is reflected at the two interference surfaces, the inner surface of the quartz backplate R2 and the inner surface of the sensitive diaphragm R1, resulting in multiple-beam interference. Owing to the low reflectivity of R1 and R2 (approximately 0.04) [19], the reflected spectrum can be approximated using a two-beam interference model, and its intensity can be expressed as follows [6,20]:

(1) $$I=I_{1}+I_{2}+2\sqrt{I_{1}I_{2}}\cos(\frac{4\pi nL}{\lambda }+\pi )$$

where I denotes the intensity of the interference spectrum, I_1_ and I_2_ denote the intensities of the two reflected beams from the interference surfaces R1 and R2, respectively, n is the refractive index of the medium inside the FP cavity, L is the cavity length of the FP cavity, *λ* is the wavelength of the light, and *π* represents the additional phase introduced by the half-wave loss. As indicated by Equation (1), the reflectivity of the interference surfaces has a significant influence on the optical signal of the sensor [21]. Since the reflective surfaces R1 and R2 serve as the two interference surfaces of the sensor’s FP cavity, their surface roughness can affect the quality of the optical interference signal. Therefore, reducing the reflectivity of the outer surface of the backplate while minimizing optical losses during signal transmission is essential for improving the quality of the sensor’s optical signal.

When the pressure-sensitive diaphragm deforms under the applied external pressure, the cavity length L of the FP cavity changes accordingly. According to the small deflection theory, the central deflection of the circular diaphragm can be expressed as follows [8,22]:

(2) $$y=\frac{3(1-\mu^{2})P}{16Eh^{3}}r^{4}$$

where *μ* is the Poisson’s ratio of the diaphragm material, E is Young’s modulus, P is the applied external pressure, h is the diaphragm thickness, and r is the effective radius of the diaphragm. As indicated by Equation (2), the applied external pressure induces a change in the cavity length of the FP cavity, and P is linearly related to the change in the cavity length. According to Equation (1), the change in the cavity length results in a variation in the optical interference signal of the sensor. By acquiring and processing the optical signal of the sensor, the variation in the cavity length L can be determined, thereby enabling the measurement of changes in the applied external pressure.

The sensitivity, pressure measurement range, and natural frequency of the all-quartz pressure sensor are determined by the effective radius and thickness of the quartz diaphragm [23,24]. The sensitivity of the sensor increases with increasing effective diaphragm radius and decreasing diaphragm thickness. However, an excessively large effective radius or an excessively small diaphragm thickness can reduce the pressure measurement range of the sensor. Therefore, the overall dimensions, sensitivity, and pressure measurement range should be considered comprehensively during sensor design. By appropriately tailoring the radius and thickness of the diaphragm, the desired overall dimensions, pressure measurement range, and sensitivity of the all-quartz pressure sensor can be achieved. For the all-quartz pressure sensor designed in this study, the effective diaphragm diameter is 2.5 mm and the diaphragm thickness is 45 μm, corresponding to a theoretical pressure sensitivity of 67.3 nm/kPa, as summarized in Table 1.

## 3. Sensor Fabrication

The fabrication procedure of the all-quartz fiber-optic Fabry–Pérot pressure sensor based on MEMS technology mainly consists of the wet etching of the quartz sensing diaphragm, through-hole machining of the quartz cavity layer and quartz substrate, preparation of the nanoforest array on the quartz backplate, and wafer bonding and dicing, as well as integration between the sensing element and optical fiber. The quartz sensing diaphragm is fabricated from a 2-inch quartz wafer with a thickness of 200 μm; the quartz cavity layer is prepared using a 2-inch, 100 μm-thick quartz wafer; the quartz backplate is produced from a 2-inch quartz wafer of 400 μm in thickness; and the quartz substrate is manufactured with a 2-inch quartz wafer of 2 mm thickness.

**Figure 2:**
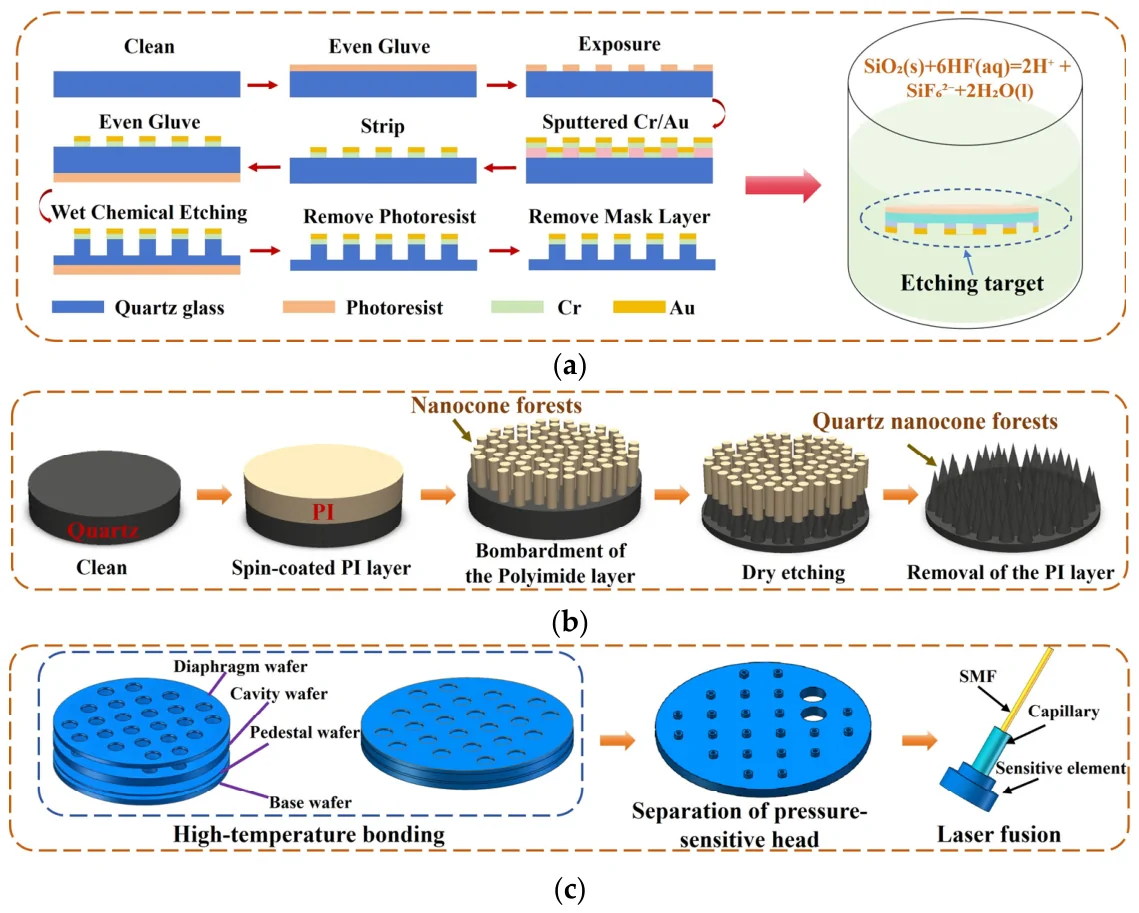
(**a**) Wet etching of the sensing diaphragm. (**b**) Fabrication of the nanoforest array. (**c**) Dicing of the sensing element and its integration with the optical fiber.

The quartz sensing diaphragm is fabricated by wet etching quartz wafers using hydrofluoric acid (HF), and the detailed process flow is illustrated in Figure 2a. First, the quartz wafer is cleaned, and then photoresist is spin-coated on its front side. After baking, exposure and development are performed sequentially. Subsequently, plasma ashing is adopted to treat the exposed wafer surface for thorough removal of residual photoresist and surface contaminants, so as to guarantee the cleanliness of subsequent processing regions. Thereafter, a 100 nm-thick chromium (Cr) protective layer and a 500 nm-thick gold (Au) protective layer are sequentially sputter-deposited on the wafer surface, and then a lift-off process is performed. The non-etching regions are masked by a metallic grinding layer. Meanwhile, photoresist is spin-coated on the back side of the quartz wafer and baked to prevent the back side from being corroded by HF solution in subsequent etching. During etching, the quartz wafer is immersed in HF solution with its etching face facing downward for wet etching. Once the wafer thickness reaches the designed target value, the metallic grinding protective layer is stripped off, and a quartz sensing diaphragm with predefined thickness is finally obtained. This standardized fabrication process has been reported in detail in our previous work [25]. The proposed method realizes wafer-level fabrication of quartz wafers based on MEMS processes and delivers favorable processing uniformity with a machining precision of ≤1 μm. The through-holes in the quartz cavity layer and quartz substrate are fabricated via CNC machining with a machining precision of ≤0.01 mm, which satisfies the structural dimension and assembly accuracy requirements of pressure-sensitive components. The fabrication process of the nanoforest array on the quartz backplate is illustrated in Figure 2b. First, a polyimide (PI) film is spin-coated onto the quartz substrate, then the PI film is subjected to oxygen plasma bombardment. The specific process parameters are set as follows: oxygen flow rate: 120 sccm, chamber pressure: 5 Pa, radio-frequency (RF) power: 200 W, and treatment duration: 30 min. After the above treatment, a densely distributed nanofiber forest structure is formed on the quartz substrate surface. This vertically oriented nanofiber array serves as a nanomask for the subsequent dry etching procedure. Using the obtained nanofiber array as the mask, reactive ion etching (RIE) is adopted to perform anisotropic etching on the quartz substrate, generating a dual-layer forest structure consisting of nanofibers and quartz nanocones. The RIE etching parameters are configured as follows: mixed-gas flow rates of SF_6_/CHF_3_/He equal to 5/32/150 sccm, RF power: 200 W, chamber pressure: 1850 mTorr, and etching time: 6 min. Upon etching completion, the sample is rinsed at room temperature using buffered oxide etchant (BOE) with an NH_4_F-to-HF volume ratio of 7:1 for 5 s to thoroughly remove residual nanofiber masks, yielding a pure-quartz nanocone forest array. The above-described nanoforest array fabrication method accomplishes quartz wafer etching based on MEMS technology. No foreign materials are introduced throughout the whole procedure. Accordingly, adverse effects on the high-temperature stability of devices, such as thermal expansion coefficient mismatch between dissimilar materials, can be effectively suppressed, endowing the resulting quartz nanoforest structure with outstanding high-temperature operational stability. After fabricating all functional structures, the quartz sensing diaphragm, quartz cavity layer, quartz backplate, and quartz substrate are stacked in a top-down sequence, where the upper surface of the quartz backplate faces the inner surface of the quartz sensing diaphragm. Following precise wafer alignment, high-temperature thermocompression bonding is employed to achieve monolithic interconnection of all quartz layers with a bonding temperature of 1000 °C, bonding pressure of 4 MPa, and holding time of 140 min, as shown in Figure 2c. Subsequently, the bonded quartz wafer is diced. Carbon dioxide laser machining is utilized for adhesive-free integration of the all-quartz pressure-sensitive element, quartz capillary, and gold-coated quartz single-mode optical fiber.

**Figure 3:**
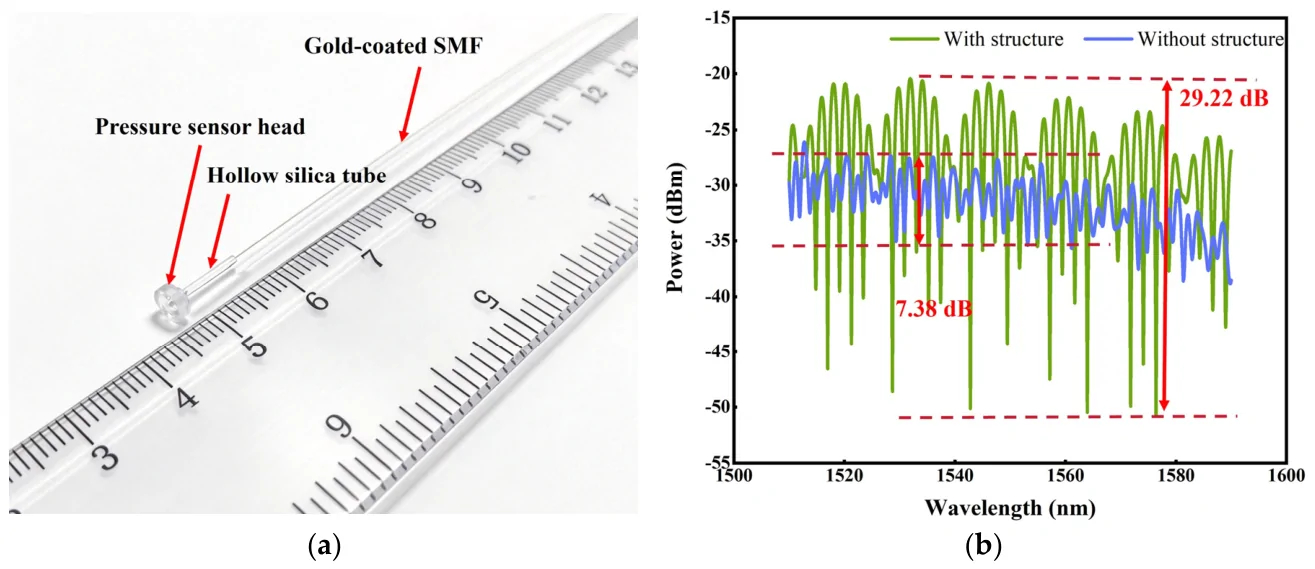
(**a**) Photograph of the fabricated sensor. (**b**) Comparison of spectral results.

The all-quartz pressure-sensitive component is obtained, and its photograph is presented in Figure 3a. To verify the optical interference signal quality of the fabricated all-quartz fiber-optic FP high-temperature pressure sensor, output spectral tests were conducted on the pressure-sensitive component integrated with a nanoforest array and quartz microsphere lens, as well as on a control device without the above integrated structures, as shown in Figure 3b. The test results show that the device integrated with the nanoforest array and quartz microsphere lens presents distinct and clear interference fringes with a maximum peak-to-valley difference of 29.22 dB. In contrast, the control device without the aforementioned structures exhibits a maximum peak-to-valley difference of only 7.38 dB, and no clear interference fringes can be observed. The peak-to-valley difference between the two devices reaches 21.84 dB, which indicates that the introduction of the nanoforest array and quartz microsphere lens can significantly improve the spectral contrast and identifiability of FP interference signals, providing a high-quality optical signal foundation for stable pressure demodulation of the sensor. During the high-temperature bonding process, the quartz wafer withstood a high temperature of 1000 °C without functional failure, which verifies the feasibility of applying the nanoforest array in high-temperature environments. Furthermore, the proposed pressure sensor adopts an all-quartz structure without introducing any heterogeneous materials during the fabrication process, which effectively avoids interfacial stress and structural failure caused by the mismatch of thermal expansion coefficients of different materials under high-temperature conditions. Therefore, the sensor possesses excellent high-temperature adaptability in terms of its structural design and material system, and can meet the application requirements of pressure measurement in high-temperature environments.

## 4. Experiment and Results

**Figure 4:**
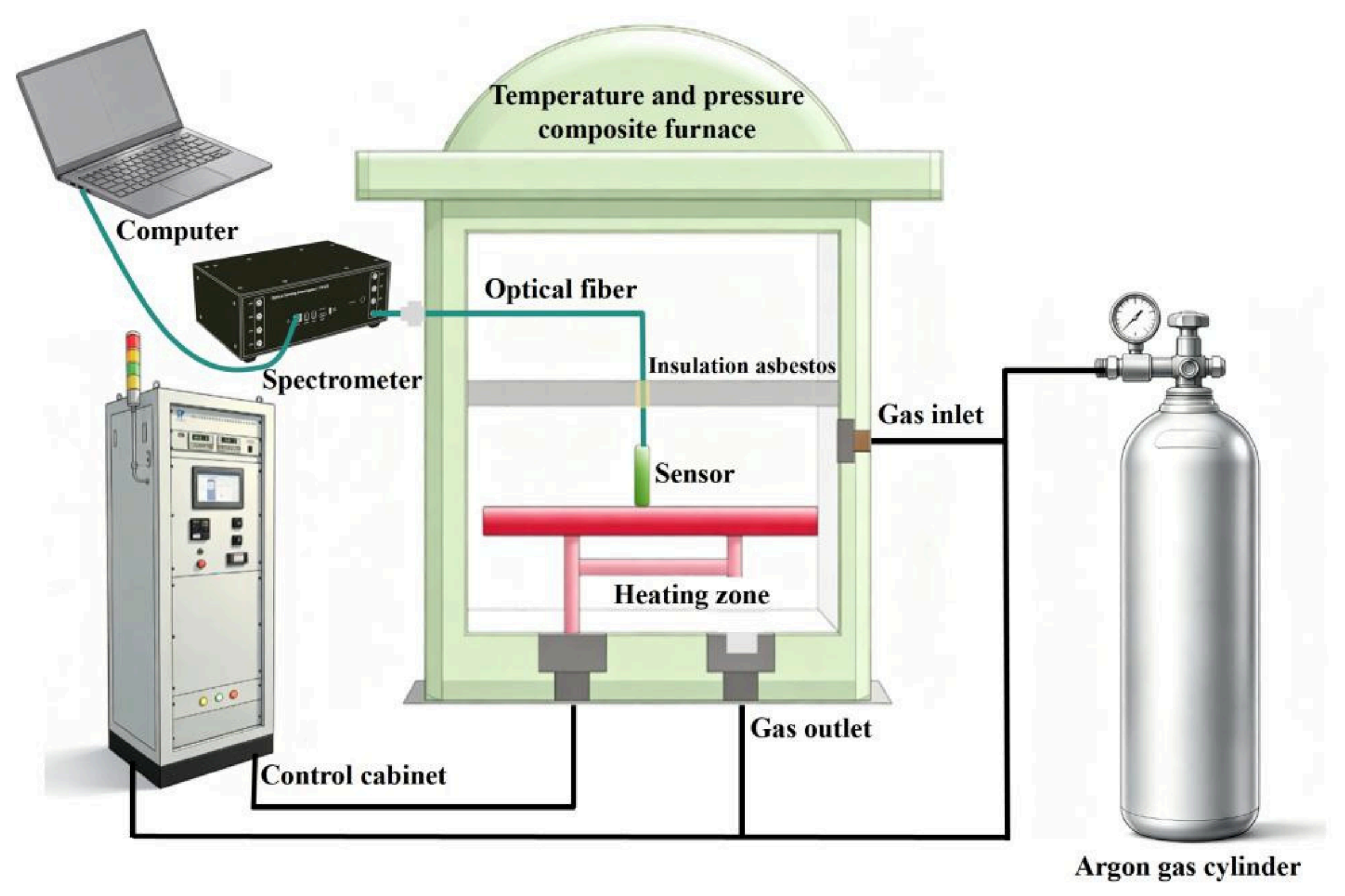
The combined temperature–pressure environmental test platform.

To evaluate the performance of the fabricated all-quartz fiber-optic FP high-temperature pressure sensor, a combined temperature–pressure environmental test platform was established, as shown in Figure 4. The test system mainly consists of a temperature–pressure composite furnace, an argon gas cylinder, a control cabinet, an SM125 optical spectrum analyzer (Micron Optics, Inc., 1852 Century Place NE, Atlanta, GA, USA), and a computer. The temperature–pressure composite furnace provides a stable and controllable combined temperature–pressure environment for the sensor. It features a maximum operating pressure of 1 MPa and a maximum operating temperature of 1500 °C, with pressure control accuracy better than 1 kPa and temperature control accuracy better than ±1 °C. During testing, target temperature and pressure values are set via the control cabinet to precisely regulate the temperature and pressure inside the composite furnace, so that stable environmental conditions for the sensor can be realized. The tail of the sensor is connected to the optical spectrum (sm125) analyzer through a high-temperature-resistant feed-through. The optical spectrum analyzer acquires the output spectra of the sensor, and the computer records spectral data in real time throughout the test. Combined with the cross-correlation spectral demodulation algorithm, the interference spectra of the sensor under different temperature and pressure conditions are demodulated to obtain the corresponding output responses. These experimental data support the subsequent analysis of temperature–pressure sensing characteristics and the high-temperature performance of the sensor.

**Figure 5:**
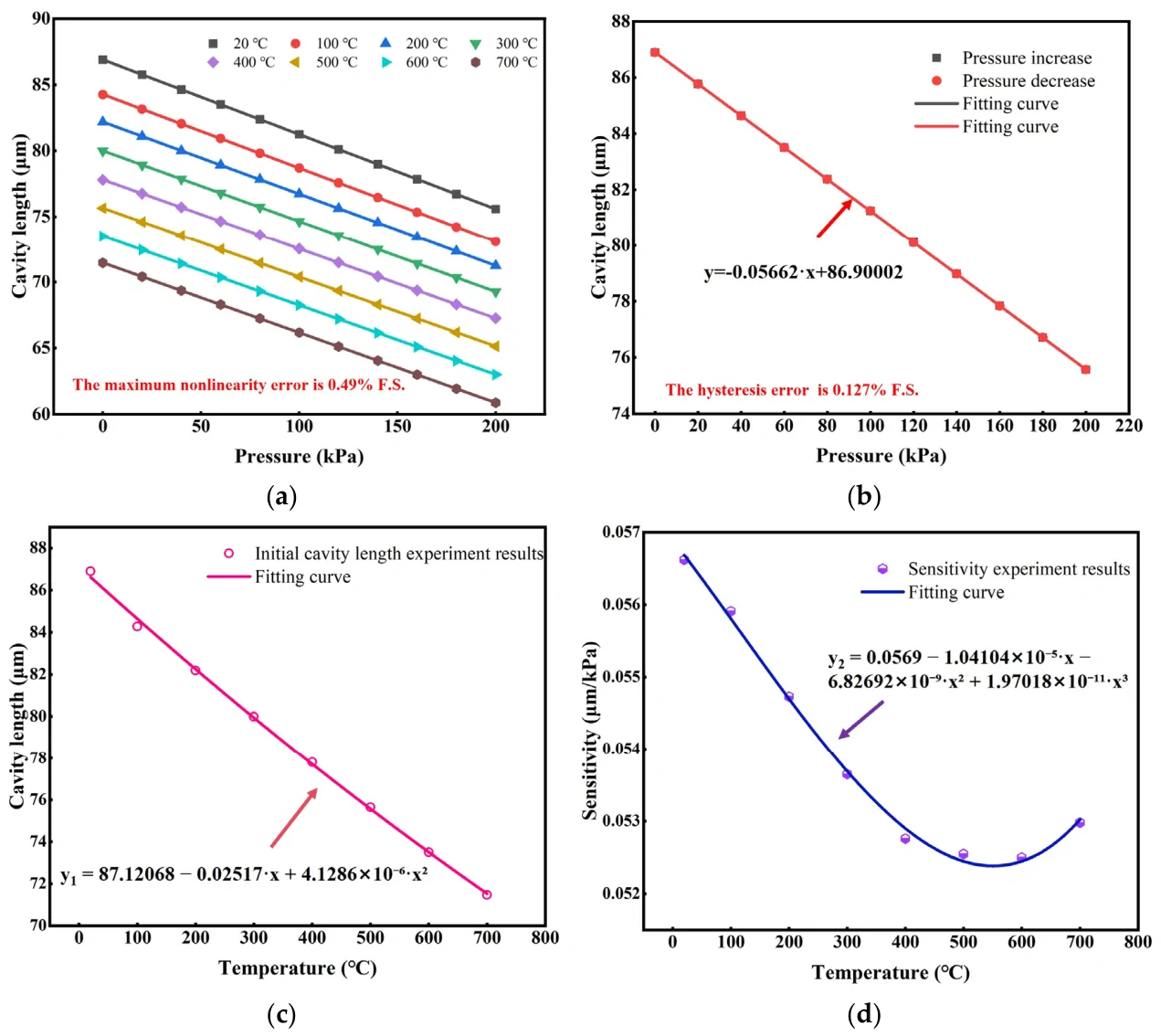
(**a**) Sensor output under different temperatures and pressures. (**b**) Hysteresis performance test of the sensor. (**c**) Initial cavity length of the sensor at various temperatures. (**d**) Sensor sensitivity at various temperatures.

To verify the pressure-sensing performance of the all-quartz fiber-optic FP high-temperature pressure sensor under high-temperature conditions, high-temperature pressure tests were performed from 20 °C to 700 °C. The test temperature was increased stepwise from 20 °C to 700 °C with a temperature step of 100 °C, and a dwell time of 20 min was applied at each temperature point to ensure the sensor reached a stable thermal equilibrium state. At each temperature point, pressure was step-loaded from 0 kPa (gauge pressure) up to 200 kPa with a pressure increment of 20 kPa. Each pressure level was held for 10 min, and the output responses of the sensor under various temperature–pressure combinations were recorded. The test results are shown in Figure 5a. Meanwhile, cyclic loading–unloading tests were carried out at room temperature, as illustrated in Figure 5b. As can be seen from Figure 5a, the all-quartz optical-fiber FP high-temperature pressure sensor exhibits favorable linear pressure response characteristics within 20–700 °C and 0–200 kPa, with a maximum nonlinear error of 0.49% F.S. Moreover, the fitted pressure response curves at different temperatures differ in initial offset and slope. The shift in the initial position mainly arises from the temperature-driven drift of FP cavity length induced by the thermal expansion behavior of quartz. As the ambient temperature rises, thermal expansion takes place inside the sensor structure, which modifies the FP cavity length and further produces temperature drift in the initial sensor output. The slope discrepancy among fitted curves at different temperatures indicates that the pressure sensitivity of the sensor is temperature-dependent. Figure 5b reveals that the sensor achieves good linear pressure responses during both loading and unloading at room temperature. The loading and unloading curves possess high consistency, yielding a hysteresis error of 0.127% F.S. The measured pressure sensitivity is 56.6 nm/kPa, which is in good agreement with theoretical predictions and demonstrates desirable pressure response performance. The slight deviation between experimental and theoretical sensitivity is mainly attributed to practical fabrication errors, such as deviations in diaphragm thickness, cavity dimensions, and other structural parameters. To further analyze the temperature effect on the sensor’s pressure-sensing performance, the initial FP cavity length and pressure sensitivity at different temperatures were extracted via fitting, as presented in Figure 5c and Figure 5d, respectively. It can be observed from Figure 5c that the initial FP cavity length decreases gradually as temperature increases, and its temperature dependence can be fitted by a quadratic function. The fitting relationship is given by y_1_ = 87.12068 − 0.02517·x + 4.1286 × 10^−6^·x^2^, where x is the temperature and y_1_ denotes the initial FP cavity length of the sensor. This variation is mainly caused by thermal expansion of structural components including the sensing diaphragm, sensing cavity, and backplate under thermal loading, and the FP cavity length change is jointly produced by the coupling effect among these structures. As shown in Figure 5d, the pressure sensitivity of the sensor exhibits obvious nonlinear characteristics with temperature. Its temperature response behavior can be fitted with a cubic function, and the fitting relationship is given by y_2_ = 0.0569 − 1.04104 × 10^−5^·x − 6.82692 × 10^−9^·x^2^ + 1.97018 × 10^−11^·x^3^, where x represents temperature and y_2_ is the pressure sensitivity of the sensor. The temperature-dependent sensitivity mainly originates from the prominent temperature dependence of Young’s modulus for quartz. As the ambient temperature varies, the elastic parameters of the material change accordingly, which further affects the deformation characteristics of the sensing diaphragm under pressure and the variation of FP cavity length, and ultimately results in the shift of the sensor’s pressure sensitivity.

**Figure 6:**
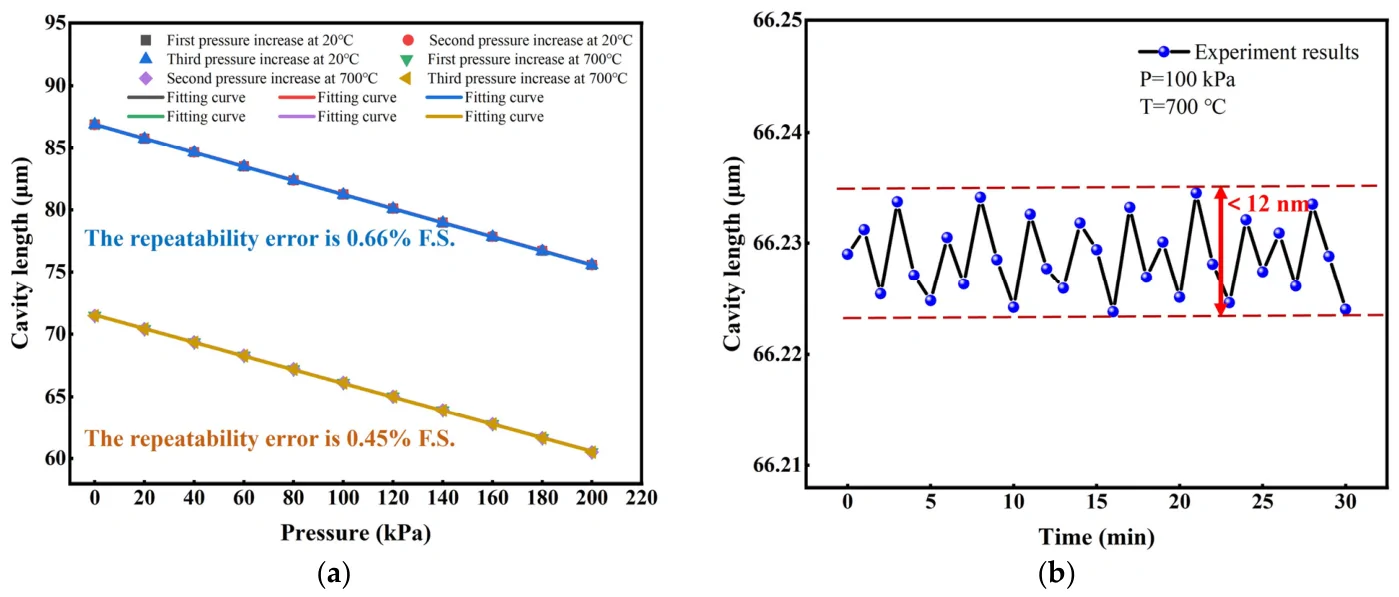
(**a**) Results of three repeated pressure-loading tests at room temperature and 700 °C. (**b**) High-temperature stability test result.

To evaluate the operational reliability and repeatability of the sensor at room temperature and high temperature, three repeated pressure-loading tests were carried out at room temperature and 700 °C, respectively. The test results are presented in Figure 6. As shown in Figure 6a, the sensor exhibits favorable linear pressure responses during the three loading cycles at both room temperature and 700 °C, and the pressure response curves of each test show high consistency. The repeatability error is 0.66% F.S. at room temperature and 0.45% F.S. at 700 °C. To further assess the long-term stability of the sensor at 700 °C, a pressure of 100 kPa was applied to the sensor and maintained for 30 min at 700 °C. The sensor output was recorded every minute, and the corresponding results are shown in Figure 6b. It can be observed from Figure 6b that the output fluctuation of the sensor is less than 12 nm under 100 kPa at 700 °C. This demonstrates the favorable operational stability of the sensor at high temperature, and no functional failure occurs for the proposed sensor structure under high-temperature conditions. The above results reveal that the sensor possesses excellent repeatability and stable pressure response characteristics at both room temperature and 700 °C, which satisfies the application requirements for pressure measurement in high-temperature environments.

## 5. Conclusions

In this paper, an all-quartz fiber-optic FP high-temperature pressure sensor is fabricated by combining MEMS technology, high-temperature bonding technology, and laser fusion welding technology. By introducing the nanoforest structure and optical microsphere lens, the quality of the optical interference signals of the sensor is effectively improved, and its high-temperature pressure-sensing performance is experimentally characterized. Experimental results demonstrate that the proposed all-quartz fiber-optic FP pressure sensor can operate stably within the temperature range of 20–700 °C. At room temperature, the sensor achieves a pressure sensitivity of 56.6 nm/kPa and a hysteresis error of 0.127% F.S. Over the full temperature range of 20–700 °C, its maximum nonlinear error is 0.49% F.S. The repeatability errors are 0.66% F.S. and 0.45% F.S. at 20 °C and 700 °C, respectively, which indicates a favorable linear pressure response and repeatability of the sensor. Under 700 °C and 100 kPa, the fluctuation amplitude of the sensor output cavity length is less than 12 nm, showing excellent high-temperature stability. The above results verify that the nanoforest array and optical microsphere lens can maintain stable optical modulation and signal transmission performance even at 700 °C. In this work, the nanoforest array is introduced into optical-fiber FP high-temperature pressure sensors, and its feasibility for high-temperature pressure measurement is validated experimentally. The proposed all-quartz fiber-optic FP pressure sensor features a compact structure, low cost, easy batch manufacturing, and high device consistency. It provides a feasible strategy to improve the interference signal quality of fiber-optic pressure sensors for high-temperature environments, and holds great application potential for pressure measurement in high-temperature and other extreme environments.

## Figures and Tables

**Table 1:** Sensor and component size table.

Description	Symbol	Value
Diaphragm radius	r	1.25 mm
Diaphragm thickness	h	45 μm
Diaphragm Young’s modulus	E	72.5 GPa
Diaphragm Poisson’s ratio	*µ*	0.17
Pressure range	P	200 kPa
Theoretical sensitivity	-	67.3 nm/kPa

## Data Availability

The original contributions presented in this study are included in the article. Further inquiries can be directed to the corresponding author.
